# Metabolic syndrome in stable long-term antipsychotic monotherapy and polypharmacy among patients with schizophrenia: a real-world comparative study

**DOI:** 10.3389/fpsyt.2026.1909155

**Published:** 2026-10-07

**Authors:** Haluk Usta, Didem Beşikci Keleş

**Affiliations:** Department of Psychiatry, University of Health Sciences Erenköy Training and Research Hospital for Mental and Neurological Disorders, İstanbul, Türkiye

**Keywords:** antipsychotic polypharmacy, cardiometabolic risk, clozapine, metabolic syndrome, olanzapine, real-world study, schizophrenia, smoking

## Abstract

**Background:**

Metabolic syndrome is a major contributor to premature cardiovascular morbidity and mortality among people with schizophrenia. Antipsychotic polypharmacy is common, but its independent metabolic burden remains uncertain.

**Objective:**

To compare metabolic syndrome in patients receiving stable long-term antipsychotic monotherapy or polypharmacy and to examine high metabolic-risk exposure, chlorpromazine-equivalent dose, and long-acting injectable (LAI) use.

**Methods:**

This retrospective study included 370 adults with schizophrenia spectrum disorders receiving an unchanged antipsychotic regimen for at least five years at a community mental health center. Patients received monotherapy (n=144) or polypharmacy (n=226). Metabolic syndrome was defined using International Diabetes Federation criteria. Antipsychotic dose was standardized to chlorpromazine equivalents, and multivariable logistic regression was used.

**Results:**

Metabolic syndrome was present in 143 patients (38.6%) and was numerically more frequent with polypharmacy than monotherapy (41.2% vs. 34.7%, p=0.216). LAI use (67.3% vs. 37.5%, p<0.001) and median chlorpromazine-equivalent dose (1010.0 vs. 454.5 mg/day, p<0.001) were higher with polypharmacy. However, neither LAI use (adjusted OR = 1.220, 95% CI 0.767-1.941, p=0.401) nor chlorpromazine-equivalent dose per 100 mg/day (adjusted OR = 1.003, 95% CI 0.954-1.055, p=0.899) was independently associated with metabolic syndrome. Current smoking remained associated with metabolic syndrome (adjusted OR = 1.996, 95% CI 1.251-3.185, p=0.004).

**Conclusions:**

In this selected cohort with stable long-term treatment, polypharmacy was associated with greater dose and LAI exposure, but independent associations of treatment modality, current dose, LAI use, or olanzapine/clozapine exposure with metabolic syndrome were not detected. Comprehensive cardiometabolic monitoring and smoking cessation remain priorities.

## Introduction

Schizophrenia is a chronic and disabling psychiatric disorder associated with substantial functional impairment and reduced life expectancy. Although antipsychotic medications remain the cornerstone of treatment, people with schizophrenia have markedly increased cardiovascular morbidity and premature mortality compared with the general population, and cardiometabolic disorders are among the leading contributors to this excess mortality burden (1).

Metabolic syndrome, characterized by the clustering of central obesity, dyslipidemia, impaired glucose metabolism, and hypertension, is highly prevalent in patients with schizophrenia. Large meta-analytic evidence indicates that approximately one-third of individuals with severe mental illness meet criteria for metabolic syndrome, with rates substantially exceeding those observed in the general population (2). Multiple factors contribute to this elevated cardiometabolic risk, including sedentary lifestyle, smoking, poor dietary habits, illness-related factors, and long-term exposure to antipsychotic medications.

Second-generation antipsychotics (SGAs) differ substantially in their metabolic liability (3). In particular, olanzapine and clozapine have consistently been associated with the greatest adverse effects on weight, glucose regulation, and lipid metabolism, whereas agents such as aripiprazole, lurasidone, and ziprasidone generally show more favorable metabolic profiles (4). These effects are clinically important because metabolic abnormalities may accumulate during long-term treatment and contribute to diabetes, cardiovascular disease, and premature mortality.

Despite guideline-based treatment approaches generally favoring antipsychotic monotherapy whenever feasible, antipsychotic polypharmacy remains common in real-world practice, particularly among patients with chronic illness, treatment resistance, severe symptoms, or recurrent hospitalization (5). Previous studies have suggested that antipsychotic polypharmacy may be associated with increased metabolic burden; however, the evidence remains inconsistent (6). A review of systematic reviews concluded that available evidence linking antipsychotic polypharmacy to metabolic syndrome is heterogeneous and generally of low quality, highlighting the need for robust real-world studies with longer treatment exposure (7).

Furthermore, many previous studies have been limited by relatively small sample sizes, short observation periods, heterogeneous diagnostic groups, or insufficient characterization of long-term antipsychotic treatment patterns (8). In particular, real-world data directly comparing stable long-term antipsychotic monotherapy and polypharmacy in schizophrenia remain limited. The independent contribution of high metabolic-risk antipsychotic exposure within polypharmacy regimens also remains insufficiently clarified.

Therefore, the present study aimed to compare the prevalence of metabolic syndrome and cardiometabolic parameters between patients with schizophrenia receiving long-term antipsychotic monotherapy and those receiving long-term antipsychotic polypharmacy in a real-world clinical setting. In addition, we investigated whether exposure to high metabolic-risk second-generation antipsychotics was associated with metabolic syndrome among chronically treated patients with schizophrenia.

## Methods

### Study design and setting

This retrospective comparative observational study was conducted at the Kadıköy Community Mental Health Center at Erenköy Mental and Neurological Diseases Training and Research Hospital. Medical records of patients diagnosed with schizophrenia spectrum disorders were reviewed to investigate the association between long-term antipsychotic treatment strategies and metabolic syndrome.

### Ethics

The study protocol was approved by the Scientific Research Ethics Committee of Erenköy Mental and Neurological Diseases Training and Research Hospital (Meeting No. 2025/21, Decision No. 98, November 13, 2025). The study was conducted in accordance with the ethical principles of the Declaration of Helsinki. Because of the retrospective nature of the study and the use of anonymized medical records, informed consent was waived by the ethics committee.

### Participants

Patients were identified through the hospital electronic medical record system. All available records that met the prespecified eligibility criteria were included; no sampling or case selection was performed after eligibility was established. Individuals were eligible for inclusion if they met all of the following criteria:

- age between 18 and 65 years;
- diagnosis of schizophrenia spectrum disorder according to ICD-10 diagnostic codes (F20–F29);
- continuous antipsychotic treatment for at least five years;
- availability of metabolic assessment data, including anthropometric measurements, blood pressure recordings, and laboratory parameters within the previous 12 months.

Patients were excluded if they had:

- schizoaffective disorder or bipolar disorder;
- comorbid substance use disorder requiring active treatment;
- pregnancy;
- severe medical illness likely to affect metabolic parameters;
- incomplete clinical or laboratory records.

To ensure long-term treatment stability, antipsychotic treatment histories were reviewed using both Community Mental Health Center (CMHC) records and the national electronic health record system (e-Nabız). Patients included in the study had been followed regularly at the Kadıköy CMHC and were required to have maintained the same antipsychotic regimen for at least five years. Information from records preceding CMHC enrollment was also reviewed to confirm that no major treatment modifications had occurred before registration, including changes in antipsychotic agents, addition of new antipsychotics, or sustained dose escalation. In cases of symptom exacerbation, routine clinical practice generally consisted of increasing the dose of the existing antipsychotic, followed by short-term benzodiazepine augmentation when necessary, and, if symptoms persisted, temporary haloperidol treatment for no longer than three months. Patients requiring permanent modifications of their antipsychotic regimen were not eligible for inclusion.

The mean duration of exposure to the same active antipsychotic agent was 95 ± 26 months in the monotherapy group and 91 ± 26 months in the polypharmacy group (p = 0.17). Mean CMHC follow-up duration was similarly long in both groups (91.6 ± 27.4 vs. 92.5 ± 26.6 months, respectively). The small difference between total antipsychotic exposure duration and CMHC follow-up duration further supported the stability of treatment histories in the study cohort.

### Study size considerations

Given the retrospective chart-review design, the study size was determined by the number of available records that fulfilled the prespecified eligibility criteria rather than by a prospectively selected effect-size target. The final analytical cohort comprised 370 patients. Accordingly, the analyses emphasize effect estimates and 95% confidence intervals rather than *post hoc* power.

### Antipsychotic treatment classification

Patients were categorized according to their long-term antipsychotic treatment regimen.

- Monotherapy Group: Patients who had received the same single antipsychotic agent continuously for at least five years were classified as the monotherapy group.
- Polypharmacy Group: Patients who had received two or more antipsychotic medications concurrently as a stable treatment regimen for at least five years were classified as the polypharmacy group.

Antipsychotic medications were additionally categorized according to pharmacological class as first-generation antipsychotics (FGAs) or second-generation antipsychotics (SGAs).

To evaluate potential differences in metabolic liability, SGAs were further stratified according to established cardiometabolic risk profiles:

#### High metabolic-risk SGAs

- Clozapine
- Olanzapine

#### Moderate metabolic-risk SGAs

- Risperidone
- Paliperidone
- Quetiapine

#### Lower metabolic-risk SGAs

- Aripiprazole
- Ziprasidone
- Lurasidone

### Data collection

Demographic, clinical, anthropometric, and laboratory data were retrospectively extracted from the electronic medical records of eligible patients. Demographic and clinical variables included age, sex, educational attainment, employment status, marital status, living arrangements, smoking status, history of alcohol and substance use, family history of psychotic disorders, history of suicide attempts, and duration of illness. Anthropometric measurements comprised body weight, height, body mass index (BMI), and waist circumference. Cardiometabolic data were extracted from routine clinical and laboratory records and included the presence of diabetes mellitus, hypertension, hypertriglyceridemia, low HDL cholesterol, central obesity, and metabolic syndrome components required for classification according to the International Diabetes Federation (IDF) criteria (9). Available anthropometric measurements and clinical metabolic assessments recorded during routine follow-up were also collected. Information regarding comorbid medical conditions, including diabetes mellitus, hyperlipidemia, and hypertension, was also collected. In addition, detailed information on antipsychotic treatment regimens, including the type, number, and duration of antipsychotic medications, was obtained from medical records.

During data extraction, authorized study investigators accessed the electronic medical records and assigned each eligible participant a unique study code. Direct personal identifiers, including names, national identification numbers, hospital identification numbers, contact information, and other directly identifying information, were not included in the analytical dataset. The coded dataset used for statistical analysis therefore contained no direct patient identifiers, and all analyses were performed using this de-identified dataset.

There were no missing data for the variables included in the primary analyses. Specifically, waist circumference and all variables required to determine metabolic syndrome according to the IDF criteria were available for all 370 participants. Accordingly, no imputation was performed. The completeness of the data was facilitated by the integrated national electronic health record system (e-Nabız) used in Türkiye, which enables longitudinal access to patients’ clinical and laboratory records across healthcare institutions. In addition, the Community Mental Health Center where the study was conducted maintains structured and regular clinical follow-up records, including routine anthropometric and metabolic assessments. These complementary data sources allowed the required metabolic variables to be verified for all patients included in the final analytical cohort.

### Definition of metabolic syndrome

Metabolic syndrome was defined according to the International Diabetes Federation (IDF) criteria (9). Central obesity was considered a mandatory component and was defined as a waist circumference of ≥94 cm in men and ≥80 cm in women. In addition to central obesity, the presence of at least two of the following criteria was required for the diagnosis of metabolic syndrome: triglyceride levels ≥150 mg/dL or treatment for hypertriglyceridemia; HDL cholesterol levels <40 mg/dL in men or <50 mg/dL in women; blood pressure ≥130/85 mmHg or current antihypertensive treatment; and fasting plasma glucose ≥100 mg/dL or a previous diagnosis of type 2 diabetes mellitus.

Antipsychotic treatment characteristics included treatment modality (monotherapy versus polypharmacy), duration of treatment, and exposure to high metabolic-risk second-generation antipsychotics. High metabolic-risk SGA exposure was coded as present whenever any component of the regimen was olanzapine and/or clozapine; regimens without either agent (for example, risperidone plus aripiprazole) were coded as not exposed, irrespective of the number or risk categories of the other agents.

### Antipsychotic dose and formulation exposure

Long-acting injectable exposure was coded as present when any component of the stable regimen was administered as an LAI. Total daily antipsychotic exposure was standardized to chlorpromazine-equivalent (CPZ-eq) dose using the international consensus estimates reported by Gardner et al. (20). Recorded component doses were converted and summed within each regimen. For one aripiprazole once-monthly 400-mg regimen, the dose was normalized over the 28-day administration interval (14.29 mg/day) before conversion, consistent with the approved once-monthly regimen and pharmacokinetic literature (22). DDD-based equivalents were calculated as a sensitivity analysis (21).

### Statistical analysis

Statistical analyses were performed using IBM SPSS Statistics for Mac, version 31.0 (IBM Corp., Armonk, NY, USA). The distribution of continuous variables was assessed using visual inspection of histograms and Q–Q plots together with the Shapiro–Wilk test. Normally distributed variables were presented as mean ± standard deviation (SD) and compared using the independent-samples t-test, whereas non-normally distributed variables were expressed as median (interquartile range [IQR]) and compared using the Mann–Whitney U test.

Categorical variables were summarized as frequencies and percentages and compared using Pearson’s chi-square test or Fisher’s exact test, as appropriate.

To identify factors independently associated with metabolic syndrome, variables considered clinically relevant and/or demonstrating an association in univariable analyses were entered into multivariable logistic regression models. Adjusted odds ratios (ORs) with corresponding 95% confidence intervals (CIs) were calculated.

Because body mass index (BMI) and waist circumference constitute integral components of the diagnostic criteria for metabolic syndrome, these variables were not simultaneously included in the primary multivariable model in order to minimize multicollinearity and avoid overadjustment. Model calibration was assessed using the Hosmer–Lemeshow goodness-of-fit test, and model performance was evaluated using Nagelkerke’s R².

Exposure analyses compared LAI proportions using Pearson’s chi-square or Fisher’s exact test and compared CPZ-eq distributions using the Mann-Whitney U test. Holm-adjusted p-values were calculated as a sensitivity check for the four new dose/route comparisons. In addition to the original clinical model, a complementary exposure model replaced the correlated treatment-pattern indicator with CPZ-eq dose (per 100 mg/day) and LAI use and adjusted for age, sex, smoking, and olanzapine/clozapine exposure. Age-illness-duration correlation and variance inflation factors were examined; illness duration replaced age in a sensitivity model. Complete-case analysis was used, and all 370 participants had complete data for these analyses.

All statistical tests were two-tailed, and a p-value <0.05 was considered statistically significant.

## Results

### Participant characteristics

A total of 370 patients with schizophrenia who had remained on a stable antipsychotic regimen for at least five years were included in the study. Of these, 144 (38.9%) were receiving antipsychotic monotherapy and 226 (61.1%) were receiving antipsychotic polypharmacy. Overall, 207 participants (55.9%) were male and 163 (44.1%) were female. The mean age of the study population was approximately 49 years.

Several sociodemographic characteristics differed between treatment groups. Patients receiving polypharmacy were more frequently male than those receiving monotherapy (60.2% vs. 49.3%, p=0.040). Significant differences were also observed in employment status (p<0.001). Individuals receiving polypharmacy were more likely to be unemployed (60.2% vs. 42.4%) or medically retired because of disability (10.2% vs. 4.2%), whereas active employment was more common among patients receiving monotherapy (46.5% vs. 24.3%).

Marital status differed significantly between groups (p=0.007). Patients receiving polypharmacy were more frequently single (74.8% vs. 61.8%), whereas marriage was more common among those receiving monotherapy (20.8% vs. 9.7%). Educational attainment, social support, socioeconomic status, living arrangements, family history of psychosis, smoking status, alcohol use history, and substance use history did not significantly differ between groups (all p>0.05).

A history of suicide attempts was significantly more prevalent among patients receiving polypharmacy than among those receiving monotherapy (45.6% vs. 30.6%, p=0.004) (**Table 1**).

**Table 1:** Demographic, clinical, and anthropometric characteristics of patients receiving long-term antipsychotic monotherapy and polypharmacy.

Variable	Monotherapy (n=144)	Polypharmacy (n=226)	p value
Age, years	48 ± 10	48 ± 10	0.819
Male sex, n (%)	71 (49.3)	136 (60.2)	0.040*
Smoking, n (%)	80 (55.6)	138 (61.1)	0.216
Alcohol use history, n (%)	17 (11.8)	26 (11.5)	0.972
Substance use history, n (%)	12 (8.3)	25 (11.1)	0.309
Family history of psychosis, n (%)	84 (58.3)	133 (58.8)	0.748
History of suicide attempt, n (%)	44 (30.6)	103 (45.6)	0.004*
Age at illness onset, years	28 ± 10	24 ± 7	<0.001*
Duration of illness, months	252 ± 111	302 ± 118	<0.001*
Duration of treatment, months	95 ± 26	91 ± 26	
Height, cm	168 ± 10	170 ± 10	0.179
Weight, kg	81 ± 16	84 ± 16	0.072
BMI, kg/m²	28.7 ± 5.1	29.4 ± 5.3	0.252
Waist circumference, cm	100 ± 12	103 ± 13	0.026*

Bold values and asterisks (*) indicate statistical significance at p < 0.05.

### Clinical and anthropometric characteristics

Patients receiving antipsychotic polypharmacy exhibited an earlier age at illness onset and a longer duration of illness compared with those receiving monotherapy. The mean age at illness onset was 24.0 ± 7.0 years in the polypharmacy group and 28.0 ± 10.0 years in the monotherapy group (p < 0.001). Correspondingly, the mean duration of illness was significantly longer among patients receiving polypharmacy (302 ± 118 months) than among those receiving monotherapy (252 ± 111 months; p < 0.001).

Chronological age did not differ between treatment groups (48 ± 10 vs. 48 ± 10 years, p = 0.819). Similarly, body height, body weight, and body mass index were comparable between groups (all p > 0.05). However, patients receiving polypharmacy demonstrated a significantly greater waist circumference than those receiving monotherapy (103 ± 13 cm vs. 100 ± 12 cm, p = 0.026) (**Table 1**).

### Metabolic syndrome prevalence

Overall, 143 of the 370 participants (38.6%) met the International Diabetes Federation criteria for metabolic syndrome. The prevalence of metabolic syndrome was numerically higher among patients receiving antipsychotic polypharmacy than among those receiving monotherapy (41.2% vs. 34.7%). However, this difference did not reach statistical significance (χ²=1.53, p=0.216) (**Figure 1**).

**Figure 1:**
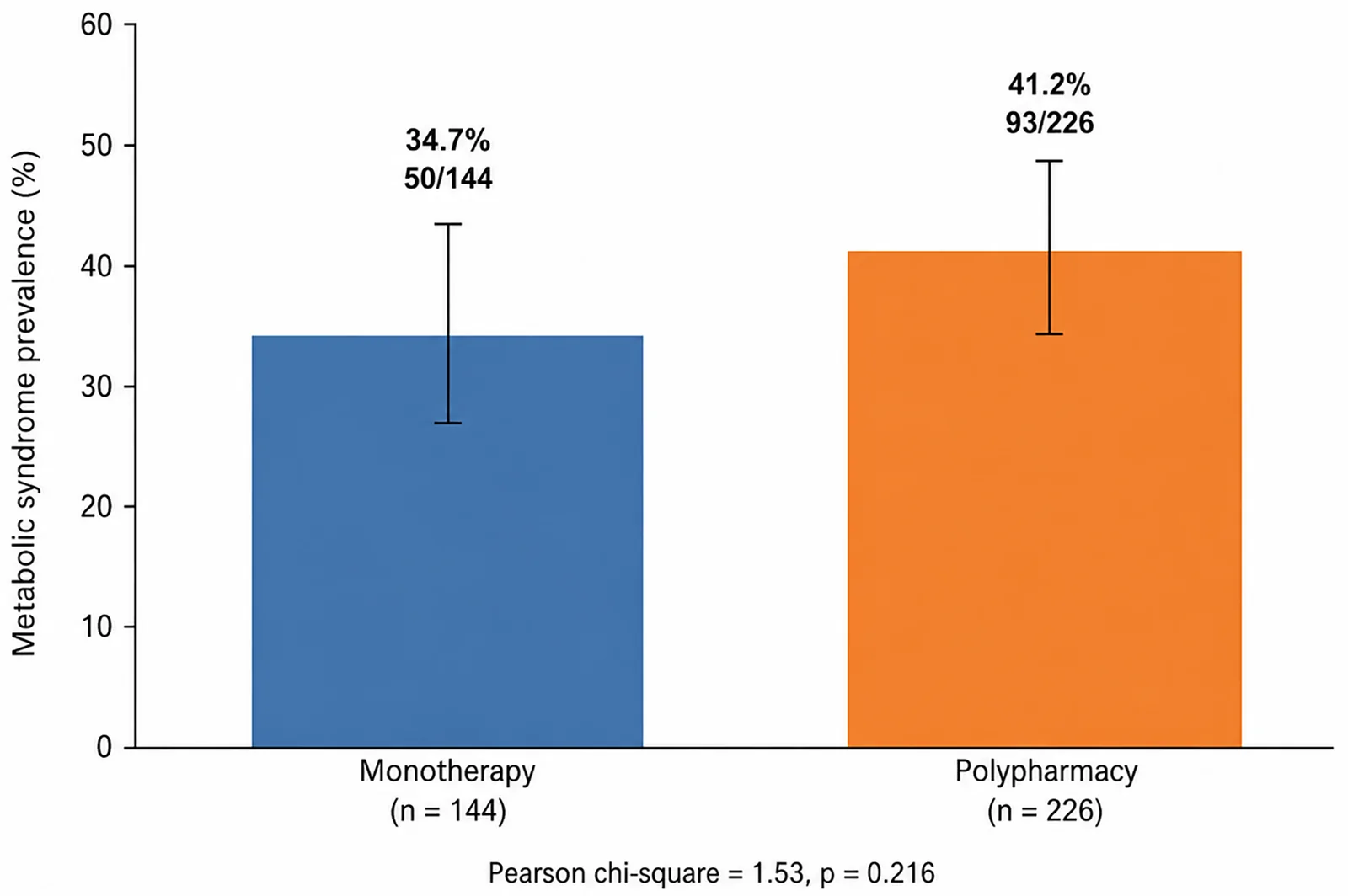
Metabolic syndrome prevalence according to antipsychotic treatment strategy. Prevalence of metabolic syndrome among patients receiving antipsychotic monotherapy and antipsychotic polypharmacy. Error bars represent 95% confidence intervals. Although metabolic syndrome was numerically more frequent among patients receiving polypharmacy (41.2%) than among those receiving monotherapy (34.7%), the difference was not statistically significant (Pearson χ² = 1.53, p = 0.216).

### Cardiometabolic characteristics

The prevalence of individual cardiometabolic abnormalities was broadly comparable between the monotherapy and polypharmacy groups. Diabetes mellitus was observed in 28 patients (19.4%) receiving monotherapy and 51 patients (22.6%) receiving polypharmacy. Hypertension was present in 21 patients (14.6%) in the monotherapy group and 27 patients (11.9%) in the polypharmacy group. Elevated triglyceride status was identified in 50.0% of patients receiving monotherapy and 48.2% of those receiving polypharmacy, while low HDL cholesterol was observed in 43.1% and 46.5% of patients, respectively. Central obesity was highly prevalent in both groups, affecting 87.5% of patients receiving monotherapy and 84.5% of those receiving polypharmacy.

As noted above, metabolic syndrome was present in 34.7% of the monotherapy group and 41.2% of the polypharmacy group; however, this between-group difference was not statistically significant (**Table 2**).

**Table 2:** Cardiometabolic and antipsychotic exposure characteristics.

Variable	Monotherapy (n=144)	Polypharmacy (n=226)	p value
Diabetes mellitus, n (%)	28 (19.4)	51 (22.6)	0.431
Hypertension, n (%)	21 (14.6)	27 (11.9)	0.334
Hypertriglyceridemia, n (%)	72 (50.0)	109 (48.2)	0.614
Low HDL cholesterol, n (%)	62 (43.1)	105 (46.5)	0.490
Central obesity, n (%)	126 (87.5)	191 (84.5)	0.384
Metabolic syndrome, n (%)	50 (34.7)	93 (41.2)	0.216
LAI use, n (%)	54 (37.5)	152 (67.3)	<0.001*
CPZ-eq dose, mg/day, median [IQR]	454.5 [304.5-606.0]	1010.0 [757.5-1259.2]	<0.001*
High-risk SGA exposure†, n (%)	54 (37.5)	180 (79.6)	<0.001*

^†^Exposure to olanzapine and/or clozapine.

Bold values and asterisks (*) indicate statistical significance at p < 0.05.

### Antipsychotic treatment patterns

Among patients receiving antipsychotic polypharmacy, the majority were treated with two antipsychotic agents (n=174, 77.0%), followed by three agents (n=45, 19.9%) and four agents (n=7, 3.1%). The polypharmacy regimens were heterogeneous and included various combinations of second-generation antipsychotics, first-generation antipsychotics, and mixed first- and second-generation antipsychotic regimens. Clozapine- and olanzapine-containing combinations were frequently represented among the polypharmacy regimens.

The distribution and chlorpromazine-equivalent doses of individual antipsychotic medications are presented in **Table 3**. Olanzapine was the most frequently used agent (n=144, 38.9%), followed by paliperidone (n=104, 28.1%), aripiprazole (n=97, 26.2%), and clozapine (n=95, 25.7%). Among patients receiving antipsychotic polypharmacy, individual medication categories were not mutually exclusive. The majority of patients receiving polypharmacy were treated with two antipsychotic agents, followed by three- and four-agent regimens. Consistent with the greater medication burden, total regimen-level CPZ-eq exposure was substantially higher in the polypharmacy group than in the monotherapy group (median 1010.0 [IQR 757.5–1259.2] vs. 454.5 [304.5–606.0] mg/day, p<0.001).

**Table 3:** Distribution and chlorpromazine-equivalent doses of individual antipsychotic medications in the study cohort.

Antipsychotic medication	Users, n (%)	Monotherapy, n	Polypharmacy, n	Daily dose, median [IQR], mg/day	CPZ-eq dose, median [IQR], mg/day
Olanzapine	144 (38.9)	36	108	15 [10–20]	454.5 [303.0–606.0]
Paliperidone	104 (28.1)	29	75	9 [9–12]	606.0 [606.0–808.0]
Aripiprazole	97 (26.2)	34	63	20 [15–20]	406.7 [305.0–406.7]
Clozapine	95 (25.7)	18	77	400 [300–500]	606.0 [454.5–757.5]
Risperidone	69 (18.6)	18	51	6 [4–6]	606.0 [404.0–606.0]
Quetiapine	56 (15.1)	2	54	300 [200–450]	245.4 [163.6–368.1]
Zuclopenthixol	37 (10.0)	3	34	10 [10–10]	100.0 [100.0–100.0]
Amisulpride	27 (7.3)	2	25	400 [400–800]	351.5 [351.5–703.0]
Haloperidol	21 (5.7)	1	20	10 [10–20]	606.0 [606.0–1212.0]
Sulpiride	5 (1.4)	1	4	400 [200–400]	303.0 [151.5–303.0]

Data are presented as n (%) or median [interquartile range]. Percentages were calculated using the total study cohort (N = 370) as the denominator. Individual medication categories are not mutually exclusive because patients receiving antipsychotic polypharmacy could contribute to more than one medication category. CPZ-eq values represent the chlorpromazine-equivalent contribution of each individual antipsychotic component and were calculated using the international consensus estimates of Gardner et al. For polypharmacy regimens, component CPZ-eq doses were summed to derive the total regimen-level CPZ-eq exposure. CPZ-eq, chlorpromazine-equivalent dose.

### Antipsychotic dose and LAI exposure

LAI use was more frequent with polypharmacy than monotherapy (67.3% vs. 37.5%, p<0.001). Median CPZ-eq dose was also higher with polypharmacy (1010.0 [IQR 757.5-1259.2] vs. 454.5 [304.5-606.0] mg/day, p<0.001). By contrast, LAI use did not differ significantly between patients with and without metabolic syndrome (60.1% vs. 52.9%, p=0.170), nor did CPZ-eq dose (781.7 [460.6-1110.0] vs. 727.2 [497.9-1098.9] mg/day, p=0.578).

### High metabolic-risk antipsychotic exposure

High metabolic-risk second-generation antipsychotic exposure differed significantly between the treatment groups. In the monotherapy group, 90 patients (62.5%) were treated with agents other than olanzapine or clozapine, whereas 36 (25.0%) received olanzapine and 18 (12.5%) received clozapine. In contrast, among patients receiving polypharmacy, only 46 (20.4%) were not exposed to olanzapine or clozapine, while 105 (46.5%) received olanzapine-containing regimens and 75 (33.2%) received clozapine-containing regimens.

Overall, exposure to high metabolic-risk antipsychotics was significantly more frequent in the polypharmacy group than in the monotherapy group (79.6% vs. 37.5%, χ²=68.11, p<0.001).

### High metabolic-risk antipsychotic exposure and metabolic syndrome

Exposure to high metabolic-risk second-generation antipsychotics was substantially more common among patients receiving polypharmacy than among those receiving monotherapy (79.6% vs. 37.5%, p<0.001). However, neither high-risk antipsychotic exposure nor the specific use of olanzapine or clozapine was associated with a higher prevalence of metabolic syndrome. The prevalence of metabolic syndrome was similar among patients without high-risk antipsychotic exposure (39.0%), those receiving olanzapine (39.7%), and those receiving clozapine (36.6%) (p=0.885). Likewise, when olanzapine and clozapine exposure were analyzed as a combined category, metabolic syndrome prevalence remained comparable between exposed and non-exposed patients (38.5% vs. 39.0%, p=0.923).

### Factors associated with metabolic syndrome

To identify factors independently associated with metabolic syndrome, a multivariable logistic regression analysis was performed including age, sex, duration of illness, antipsychotic treatment modality, smoking status, and high metabolic-risk antipsychotic exposure.

In the original model, which included both age and illness duration, older age was associated with higher odds of metabolic syndrome (OR = 1.042, p=0.007). Likewise, smoking was associated with an approximately twofold increase in the odds of metabolic syndrome (OR = 1.948, p=0.005).

Duration of illness showed a statistically significant but minimal association with metabolic syndrome in the multivariable model (OR = 0.997, 95% CI 0.994–1.000, p=0.020), although the magnitude of this effect was very small. Antipsychotic polypharmacy demonstrated a non-significant trend toward increased odds of metabolic syndrome (OR = 1.590, p=0.073). Neither sex (OR = 0.907, p=0.680) nor exposure to high metabolic-risk second-generation antipsychotics (OR = 0.915, p=0.729) was independently associated with metabolic syndrome (**Figure 2**).

**Figure 2:**
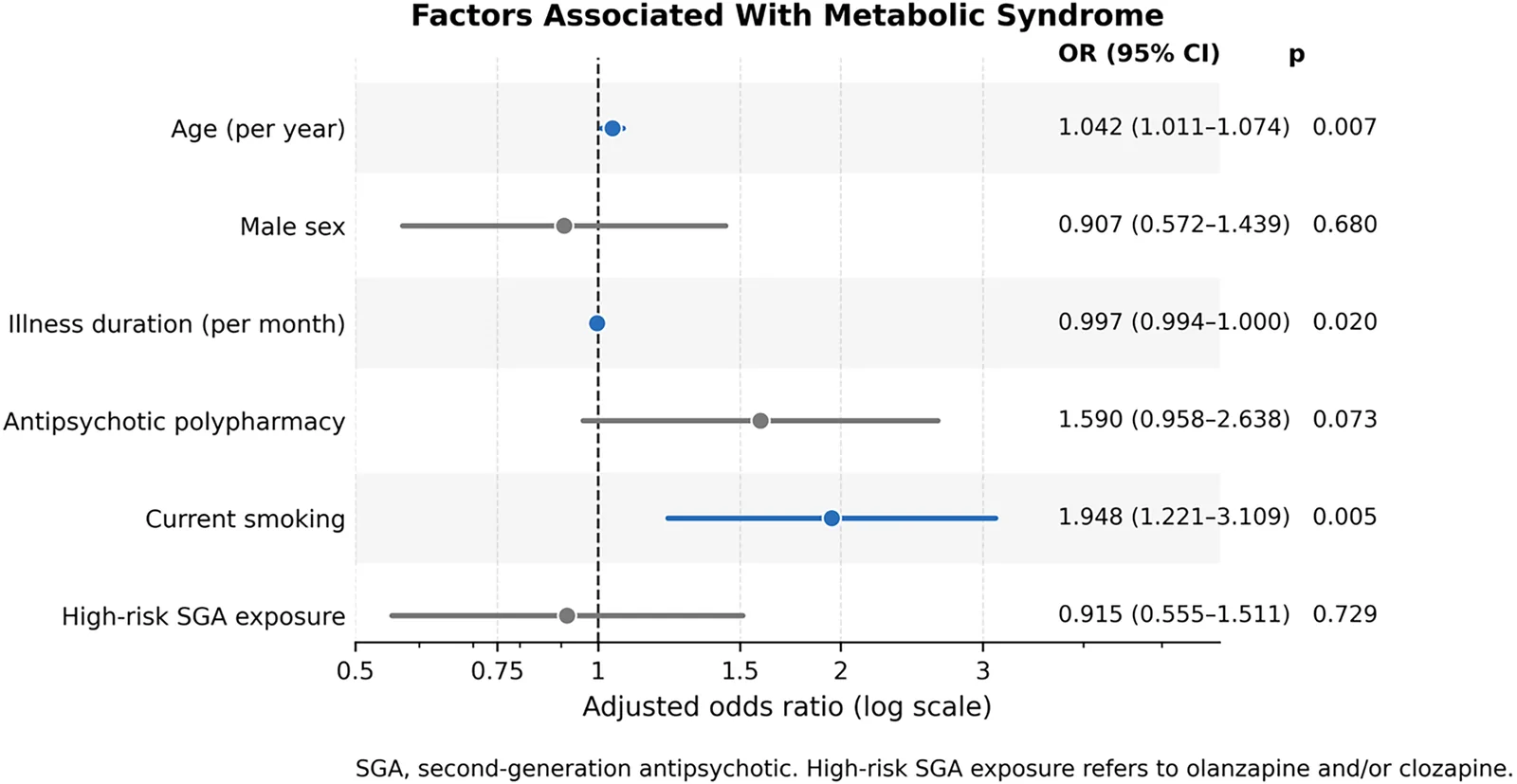
Multivariable logistic regression analysis of factors associated with metabolic syndrome. Forest plot showing adjusted odds ratios (ORs) and 95% confidence intervals (CIs) derived from the multivariable logistic regression model evaluating factors associated with metabolic syndrome. Older age and current smoking were independently associated with increased odds of metabolic syndrome, whereas sex, antipsychotic polypharmacy, and exposure to high metabolic-risk second-generation antipsychotics were not significantly associated with metabolic syndrome. High-risk second-generation antipsychotic exposure was defined as treatment with olanzapine and/or clozapine.

Age and illness duration were moderately correlated (Pearson r=0.648; Spearman rho=0.630), while the maximum variance inflation factor in the original model was 1.97. Because simultaneous inclusion materially altered the coefficients, the complementary exposure model included age without illness duration; replacing age with illness duration did not change the CPZ-eq or LAI conclusions.

### Antipsychotic burden and metabolic syndrome

To further examine antipsychotic burden, patients were stratified according to the number of concurrently prescribed agents. Metabolic syndrome prevalence was 34.7%, 40.2%, 44.4%, and 42.9% among patients receiving one, two, three, and four antipsychotics, respectively. The overall comparison was not statistically significant (Fisher’s exact p=0.588), and no significant linear trend was observed (p=0.196) (**Figure 3**).

**Figure 3:**
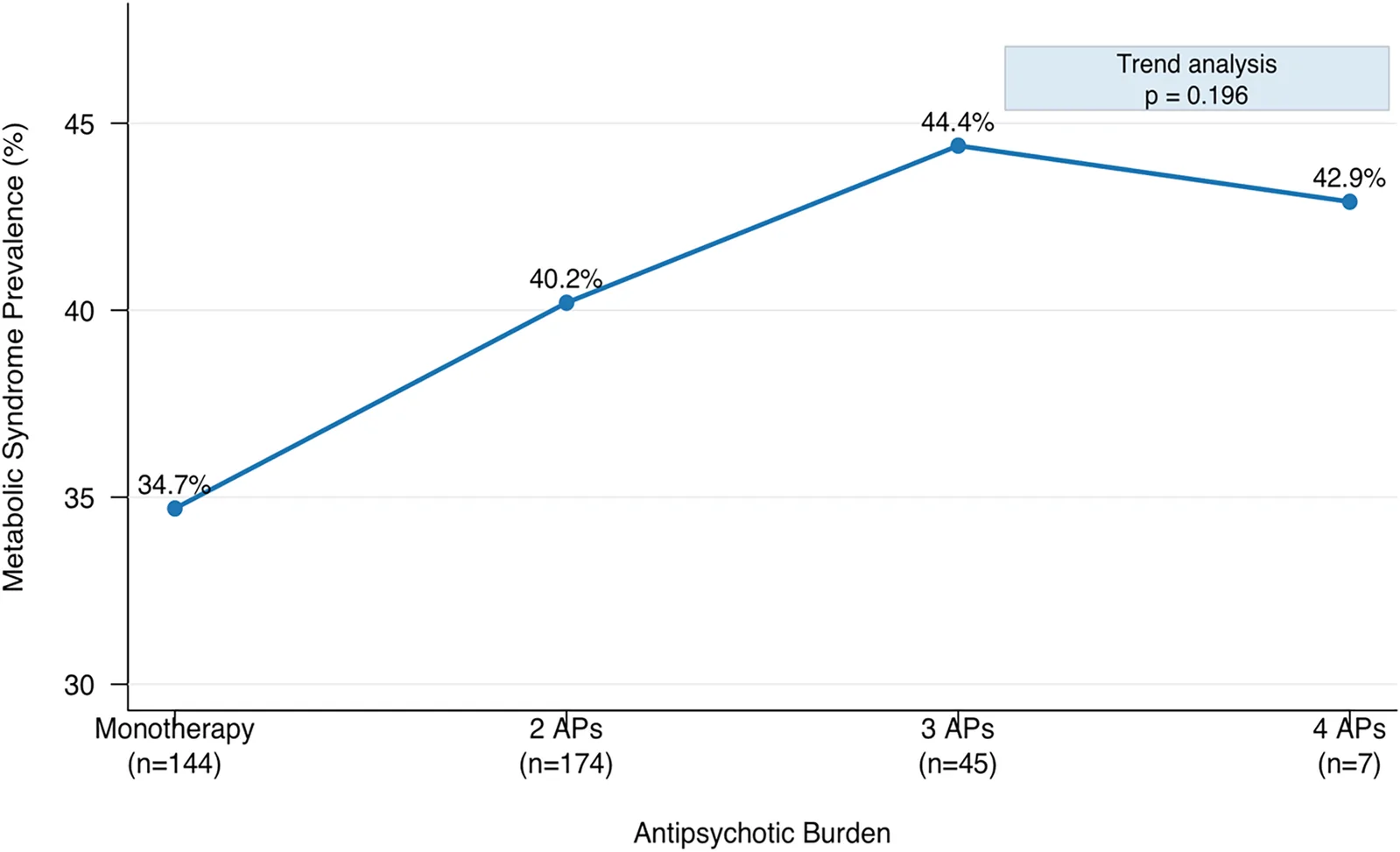
Metabolic syndrome prevalence according to antipsychotic burden. Metabolic syndrome prevalence was 34.7%, 40.2%, 44.4%, and 42.9% among patients receiving one, two, three, and four antipsychotics, respectively. Neither the overall comparison (Fisher’s exact p=0.588) nor the linear trend analysis (p=0.196) indicated a statistically significant association.

### Exploratory comparison of patients with and without metabolic syndrome

Patients with metabolic syndrome demonstrated significantly higher body weight, waist circumference, and BMI than those without metabolic syndrome (all p<0.001). Smoking was also more common among patients with metabolic syndrome (69.2% vs. 53.3%, p=0.002). In contrast, antipsychotic polypharmacy, exposure to high metabolic-risk second-generation antipsychotics, duration of illness, and history of suicide attempts did not differ significantly between groups. Although patients with metabolic syndrome had a slightly later age at illness onset, the observed difference was small and of uncertain clinical relevance (**Table 4**).

**Table 4:** Characteristics of patients with and without metabolic syndrome.

Variable	MetS (-) (n=227)	MetS (+) (n=143)	p
Age, years	47.1 ± 10.3	48.9 ± 9.2	0.101
Age at illness onset, years	24.5 ± 8.0	26.7 ± 9.0	0.017*
Duration of illness, months	285.9 ± 123.5	277.7 ± 107.3	0.516
Weight, kg	79.5 ± 15.5	89.3 ± 14.8	<0.001*
Waist circumference, cm	98.4 ± 12.1	108.6 ± 11.1	<0.001*
BMI, kg/m²	27.7 ± 4.7	31.3 ± 5.1	<0.001*
Smoking, n (%)	121 (53.3)	99 (69.2)	0.002*
High-risk SGA exposure, n (%)	144 (63.4)	90 (62.9)	0.923
Suicide attempt history, n (%)	95 (41.9)	52 (36.4)	0.294
Polypharmacy, n (%)	133 (58.6)	93 (65.0)	0.216
LAI use, n (%)	120 (52.9)	86 (60.1)	0.170
CPZ-eq dose, mg/day, median [IQR]	727.2 [497.9-1098.9]	781.7 [460.6-1110.0]	0.578

Bold values and asterisks (*) indicate statistical significance at p < 0.05.

### Complementary dose and route model

In the complementary model, neither CPZ-eq dose per 100 mg/day (adjusted OR = 1.003, 95% CI 0.954-1.055, p=0.899) nor LAI use (adjusted OR = 1.220, 95% CI 0.767-1.941, p=0.401) was independently associated with metabolic syndrome. Smoking remained associated with approximately twofold higher odds (adjusted OR = 1.996, 95% CI 1.251-3.185, p=0.004). Results were unchanged using DDD-based dose equivalents or replacing age with illness duration (**Table 5**).

**Table 5:** Complementary logistic regression model for metabolic syndrome.

Variable	B	SE	OR (Exp[B])	95% CI	p value
Age (years)	0.018	0.011	1.018	0.996-1.041	0.114
Male sex	-0.149	0.232	0.861	0.547-1.357	0.520
Current smoking	0.691	0.238	1.996	1.251-3.185	0.004*
High metabolic-risk SGA exposure	-0.017	0.245	0.983	0.608-1.590	0.945
CPZ-eq dose (per 100 mg/day)	0.003	0.026	1.003	0.954-1.055	0.899
LAI use	0.199	0.237	1.220	0.767-1.941	0.401

Dependent variable: Metabolic syndrome (yes/no). CPZ-eq, chlorpromazine-equivalent dose; LAI, long-acting injectable; SGA, second-generation antipsychotic. High-risk SGA exposure was defined as olanzapine and/or clozapine. N = 370; events=143. Omnibus chi-square=13.47, df=6, p=0.036; Hosmer-Lemeshow chi-square=7.11, df=8, p=0.525; Nagelkerke R-squared=0.049. *p<0.05.

Bold values and asterisks (*) indicate statistical significance at p < 0.05.

## Discussion

In this real-world study of patients with schizophrenia who had remained on stable antipsychotic regimens for at least five years, metabolic syndrome affected 38.6% of the cohort. Patients receiving polypharmacy had a more severe clinical and psychosocial profile, including earlier illness onset, longer illness duration, greater waist circumference, higher unemployment, and more frequent history of suicide attempts. However, metabolic syndrome was not significantly associated with treatment modality, current CPZ-eq dose, LAI use, olanzapine/clozapine exposure, or the number of antipsychotic agents. Current smoking was the most consistent independent correlate, whereas age- and illness-duration estimates were sensitive to model specification.

The overall prevalence of metabolic syndrome in this cohort was 38.6%, which is consistent with previous evidence showing a substantial cardiometabolic burden among individuals with schizophrenia and related psychotic disorders. Large meta-analytic data have demonstrated that approximately one-third of individuals with severe mental illness meet criteria for metabolic syndrome, with schizophrenia-spectrum disorders carrying particularly high rates of abdominal obesity, dyslipidemia, impaired glucose regulation, and hypertension (2, 10). This burden is clinically important because cardiovascular disease remains one of the major contributors to premature mortality in severe mental illness. A recent European Heart Journal review emphasized that patients with severe mental illness die approximately 15–20 years earlier than the general population, largely due to cardiovascular disease and sudden cardiac death (11). Therefore, the present prevalence estimate reinforces the need for systematic cardiometabolic surveillance in long-term schizophrenia care.

In Turkish cohorts, Cerit et al. reported an IDF-defined metabolic syndrome prevalence of 41% among 100 outpatients with schizophrenia or schizoaffective disorder, whereas Boke et al. reported 32% among 231 inpatients with schizophrenia (23, 24). The prevalence observed here (38.6%) lies within this local range. Differences across studies likely reflect clinical setting, age and illness-duration distributions, treatment exposure, and case mix. Together, these regional data reinforce that cardiometabolic burden is substantial in Turkish schizophrenia services.

Contrary to our initial hypothesis, antipsychotic polypharmacy was not significantly associated with metabolic syndrome. Although metabolic syndrome was numerically more frequent in the polypharmacy group than in the monotherapy group, the difference did not reach statistical significance, and polypharmacy showed only a non-significant trend in multivariable analysis. This finding should be interpreted in the context of the heterogeneous literature on antipsychotic polypharmacy (12, 13). A review of systematic reviews by Ijaz et al. concluded that the evidence linking antipsychotic polypharmacy to metabolic syndrome is conflicting and generally of low quality, with substantial heterogeneity across studies (7). More recent large-scale syntheses also indicate that antipsychotic polypharmacy is common in schizophrenia-spectrum disorders and is often associated with more severe illness, poorer functioning, hospitalization, and adverse outcomes, making confounding by indication a major methodological challenge (14). In our cohort, the polypharmacy group clearly exhibited markers of greater illness burden, yet this did not translate into a statistically significant increase in metabolic syndrome prevalence.

The absence of a significant association between olanzapine/clozapine exposure and metabolic syndrome was also noteworthy. This result may appear counterintuitive because clozapine and olanzapine are consistently recognized as antipsychotics with the highest metabolic liability. In a network meta-analysis comparing 18 antipsychotics, Pillinger et al. found substantial differences in metabolic effects across agents, with olanzapine and clozapine among those associated with the greatest adverse changes in weight and metabolic parameters (4). However, our findings suggest that, in long-term real-world cohorts, the relationship between antipsychotic metabolic liability and metabolic syndrome may not be fully captured by current exposure status alone. Several explanations are possible. First, patients receiving clozapine or olanzapine may have been more closely monitored and treated for metabolic abnormalities, attenuating group differences. Second, survivorship and selection effects may operate in long-term treated samples, whereby patients who experienced severe early metabolic deterioration may have discontinued or switched medication before inclusion. Third, cross-sectional classification of metabolic syndrome may fail to capture longitudinal changes in weight, glucose, and lipid trajectories over time.

The antipsychotic-burden analysis further supports this interpretation. Metabolic syndrome prevalence was numerically higher in each multi-agent category than with monotherapy, but neither the overall comparison nor the linear trend was statistically significant. Therefore, the number of antipsychotics alone appears to be an insufficient marker of metabolic risk. This aligns with the broader view that cardiometabolic outcomes in schizophrenia are multifactorial and arise from interactions among medication exposure, age, smoking, diet, physical inactivity, genetic vulnerability, illness chronicity, and quality of medical follow-up (11, 15). Reducing metabolic risk therefore requires structured cardiovascular risk assessment and proactive intervention throughout the treatment pathway.

The dose and formulation analyses directly address the possibility that antipsychotic burden is better represented by total exposure than by medication count. Despite a more than twofold difference in median CPZ-eq dose and much more frequent LAI use in the polypharmacy group, neither current dose nor LAI use was associated with metabolic syndrome in unadjusted or complementary adjusted analyses. These findings should not be interpreted as evidence of no metabolic effect. Rather, they indicate that a cross-sectional measure of current dose and formulation did not explain metabolic syndrome status within this highly selected, long-term stable cohort.

Age-related estimates were sensitive to model specification. In the original model that included both age and illness duration, age showed a positive association and illness duration a very small inverse association with metabolic syndrome. Because the two variables were moderately correlated and materially altered each other’s coefficients, these estimates should be interpreted cautiously. In the complementary exposure model, which included age without illness duration, age was not statistically significant. The inverse illness-duration estimate should therefore not be interpreted as a clinically meaningful protective effect.

Current smoking was the strongest modifiable factor associated with metabolic syndrome in our model, nearly doubling the odds of metabolic syndrome. Smoking is highly prevalent among people with schizophrenia and contributes substantially to cardiovascular morbidity (16). Beyond its direct vascular effects, smoking has been associated with insulin resistance, altered lipid metabolism, abdominal obesity, and systemic inflammation, all of which overlap with metabolic syndrome pathways (17). This finding has direct clinical relevance: cardiometabolic prevention in schizophrenia should integrate smoking cessation, dietary counseling, physical activity promotion, and routine metabolic monitoring rather than focusing only on antipsychotic selection (18).

A particularly interesting finding was that waist circumference was significantly greater in the polypharmacy group despite similar BMI and overall metabolic syndrome prevalence. This may indicate that central adiposity is a more sensitive marker than BMI for detecting early metabolic risk in chronically treated schizophrenia patients. Recent cardiometabolic monitoring literature also emphasizes that central obesity is often under-monitored in patients prescribed antipsychotics, despite its central role in metabolic syndrome definitions (19). Therefore, routine waist circumference measurement should be incorporated into psychiatric follow-up, especially in patients receiving complex long-term antipsychotic regimens.

Additional comparisons supported the multivariable findings. Patients with metabolic syndrome had greater body weight, waist circumference, and BMI and were more likely to smoke. In contrast, antipsychotic polypharmacy, olanzapine/clozapine exposure, LAI use, and chlorpromazine-equivalent dose did not differ significantly between metabolic syndrome groups. These findings suggest that metabolic syndrome in chronic schizophrenia may be more closely linked to traditional cardiometabolic risk factors than to current treatment configuration alone.

An additional noteworthy finding was the association between socioeconomic indicators and metabolic syndrome in the univariate analyses. Patients with metabolic syndrome tended to have lower educational attainment and less favorable socioeconomic status. Although these variables were not retained in the final multivariable model, the findings are consistent with growing evidence suggesting that social determinants of health contribute substantially to cardiometabolic risk in severe mental illness. Lower educational attainment and socioeconomic disadvantage may influence dietary quality, access to preventive healthcare, physical activity patterns, and health literacy, thereby increasing vulnerability to metabolic abnormalities. These observations further support the need for integrated cardiometabolic prevention strategies that address both clinical and social determinants of health in schizophrenia.

The present study has several strengths. First, it included a relatively large real-world cohort receiving stable long-term antipsychotic treatment. Second, treatment stability was verified using both CMHC records and the national electronic health record system, reducing exposure misclassification. Third, metabolic syndrome was defined using standardized IDF criteria. Fourth, treatment modality, high metabolic-risk SGA exposure, chlorpromazine-equivalent dose, and LAI use were examined using complementary models and dose-equivalence sensitivity analyses.

Several limitations should be acknowledged. The retrospective cross-sectional design precludes causal inference and does not allow evaluation of temporal changes in metabolic parameters. Data on diet, physical activity, cumulative lifetime antipsychotic exposure, medication adherence, lipid-lowering treatment, and detailed cardiovascular history were not available. Chlorpromazine-equivalent values reflect current prescribed doses rather than lifetime exposure and remain approximate, particularly for LAI formulations; however, findings were unchanged with a DDD-based sensitivity method. Requiring at least five years of treatment stability may have introduced survivor and selection bias because patients who previously discontinued or changed treatment after metabolic deterioration were not represented. Although the total cohort was relatively large for a single-center chart review, precision was limited in smaller treatment strata, particularly the four-antipsychotic group, and modest associations may have gone undetected. The single-center setting may limit generalizability, and residual confounding by illness severity, treatment resistance, lifestyle, and healthcare utilization cannot be excluded.

In conclusion, metabolic syndrome affected more than one-third of this selected cohort receiving stable long-term antipsychotic treatment. Although polypharmacy was associated with substantially higher total dose and more frequent LAI and olanzapine/clozapine exposure, independent associations of treatment modality, current chlorpromazine-equivalent dose, LAI use, or high metabolic-risk SGA exposure with metabolic syndrome were not detected. Smoking was the most consistent modifiable correlate. These findings support comprehensive cardiometabolic monitoring and smoking cessation across treatment strategies.

## Data Availability

The raw data supporting the conclusions of this article will be made available by the authors, without undue reservation.
